# Economic Reasoning and Interaction in Socially Extended Market Institutions

**DOI:** 10.3389/fpsyg.2019.01856

**Published:** 2019-08-19

**Authors:** Shaun Gallagher, Antonio Mastrogiorgio, Enrico Petracca

**Affiliations:** ^1^Department of Philosophy, The University of Memphis, Memphis, TN, United States; ^2^Faculty of Law, Humanities and Arts, University of Wollongong, Wollongong, NSW, Australia; ^3^Department of Neurosciences, Imaging and Clinical Sciences and CeSI-MeT, University of Chieti-Pescara, Chieti, Italy; ^4^School of Economics, Management and Statistics, University of Bologna, Bologna, Italy

**Keywords:** market structure, economic reasoning, socially extended mind, autonomy, functional integration, task dependency

## Abstract

An important part of what it means for agents to be situated in the everyday world of human affairs includes their engagement with economic practices. In this paper, we employ the concept of cognitive institutions in order to provide an enactive and interactive interpretation of market and economic reasoning. We challenge traditional views that understand markets in terms of market structures or as processors of distributed information. The alternative conception builds upon the notion of the market as a “scaffolding institution.” Introducing the concept of market as a “socially extended” cognitive institution we go beyond the notion of scaffolding to provide an enactive view of economic reasoning that understands the market participant in terms of social interactive processes and relational autonomy. Markets are more than inert devices for information processing; they can be viewed as “highly scaffolded,” where strong constraints and incentives predictably direct agents’ behavior. Building on this idea we argue that markets emerge from (a) the economic interaction of both supply and demand sides, in continual and mutual interplay, and (b) more basic social interactions. Consumer behavior in the marketplace is complex, not only contributing to determine the market price, but also extending the consumer’s cognitive processes to reliably attain a correct evaluation of the good. Moreover, this economic reasoning is socially situated and not something done in isolation from other consumers. From a socially situated, interactive point of view buying or not buying a good is something that enacts the market. This shifts the status of markets from external institutions that merely causally affect participants’ cognitive processes to social institutions that constitutively extend these cognitive processes. On this view the constraints imposed by social interactions, as well as the possibilities enabled by such interactions, are such that economic reasoning is never just an individual process carried out by an autonomous individual, classically understood. In this regard, understanding the concept of relational autonomy allows us to see how economic reasoning is always embodied, embedded in, and scaffolded by intersubjective interactions, and how such interactions make the market what it is.

## Introduction

Theories of situated cognition typically ignore an important set of situated practices that are pervasive in our everyday lives – our participation in markets and economic activities. Such activities involve the exercise of a common form of reasoning embedded in significant social interactions and/or socially and normatively defined contexts. The lack of attention to these practices, however, is for the most part one-sided. Although there is a diverse set of economists who discuss situated and institutional approaches to economic reasoning (e.g., North, 1990; Kirman, 1999; Smith, 2007; Hodgson, 2009), philosophers, psychologists and cognitive scientists who venture into the study of situated cognition or social cognitive processes rarely discuss economic reasoning or economic behavior. Moreover, when philosophers, psychologists and cognitive scientists more generally discuss economic reasoning or economic behavior it is, with few exceptions, without regard for situational or intersubjective factors. In particular, they typically frame them in terms of traditional notions of rational choice and abstract decision-making or, more recently, in terms of behavioral and cognitive biases. In addition we note that, even those who count as exceptions emphasize the idea that economic institutions, such as markets, operate primarily as external constraints on individual cognitive processes. Thereby they adopt a relatively conservative and narrow conception of situated cognition.

In contrast, we propose an enactivist interpretation of the notion of market that emphasizes the role of social interactions. On this view, markets are socially extended cognitive institutions (Gallagher, 2013). This means that market forces, rather than external constraints or inert substructures, constitute an economic order enacted in the dynamical interplay of embodied, situated, and materially engaged agents who maintain relational autonomy in a world that is both physical and social. Understanding the nature of such markets, we argue, throws light on an important dimension of everyday situated behavior.

In working out this enactive conception of market we challenge some traditional views that understand markets in terms of market structures or as processors of distributed information. To develop an alternative conception we take as a starting point the notion of the market as a ‘scaffolding institution’ understood in terms of the extended mind (Clark, 1997a, b, Chapter 9; Clark and Chalmers, 1998). Markets can be understood through the lens of *scaffolded choice* in which, rather than internal mental states – such as “beliefs, desires, or other psychological features of individuals involved” – what counts for economic reasoning are the engagements with external structures that constrain and enable agents’ behaviors and interactions (Clark, 1997a, p. 272; see also Denzau and North, 1994). In this paper we push this idea in a more enactive direction and toward a socially interactive interpretation of economic reasoning, by employing the concept of *mental* or *cognitive institution* understood in terms of the “socially extended mind” (Gallagher and Crisafi, 2009; Gallagher, 2013; Slaby and Gallagher, 2015).

We begin by reviewing some basic concepts of the market and economic reasoning, especially as understood in the neoclassical tradition and its recent developments. We do this both for purposes of later contrast, and because these neoclassical concepts influenced Andy Clark’s ideas about markets as “extended institutions,” which he proposed some 20 years ago (Clark, 1997a, b) when neoclassical models were still relatively unchallenged by behavioral economics. We then discuss Clark’s model of the market as scaffolding and constraining economic reasoning, and some deficiencies that we find in it. We next introduce the model of the socially extended mind and the concept of cognitive institution as a way to understand how markets are enacted in social interactions. This perspective allows us to see that economic reasoning is a more socially interactive process than an individual deliberation, and that in specific conditions it can veer toward purely instrumental calculation, or under different conditions promote autonomy in its relational form.

## The Traditional View of Markets: Structure, Information Processing and Mental Models

Theoretical speculations about markets as exchange and allocation mechanisms go back at least to the classic idea of the ‘invisible hand’ proposed by Adam Smith (Smith, 1776). According to this view, markets are seen as mechanisms allowing parties to exchange goods and services, thus increasing individual and social welfare. However, more recently, economists have looked at markets more generally as coordination mechanisms: whenever transaction costs – i.e., the costs of using the *price mechanism* – are low, markets are considered to solve a number of coordination problems better than alternative coordination mechanisms (see Williamson, 1981a). A “paradox” (Hodgson, 2008) in the research on markets is that economists have been nearly obsessed with market prices and market efficiency but relatively inattentive to markets as “places” in which people interact, build relationships, learn, and take care of an increasing part of their life interests. This suggests that much is still to be understood about markets. The ubiquity of the textbook definition stating that a market is a “mechanism through which buyers and sellers interact to determine prices and exchange goods, services, and assets” (Samuelson and Nordhaus, 2010, p. 26) just reinforces the sense of taken-for-grantedness surrounding this notion.

Although Samuelson and Nordhaus’s definition aptly focuses on the fundamental requirement that buyers and sellers *interact* in the market, economics has mostly refrained from studying the process of interaction itself, focusing more on the *structure* of markets and how information is processed either by market structure or by individual traders. Market structure is defined by a few key features (also called “basic conditions,” Scherer, 1980): the number, size, and distribution of buyers and sellers, market share, possibility of free entry, and product differentiation. Any particular combination of these variables is said to determine the behavior and economic decisions of market participants, and eventually market price. Ideal-typical market structures are perfect competition, monopolistic competition, oligopoly, duopoly, monopoly, monopsony, and oligopsony. More concretely, the notion of ‘market micro-structure’ aims to study the specificities of different negotiation, trading, and exchange mechanisms (O’Hara, 1995).

The work of Friedrich A. Hayek has given a fundamental epistemological twist to the study of markets by focusing on the role of market information (this has been called the “information revolution” in economics; see Mirowski and Nik-Khah, 2017). According to Hayek, information is so fragmentary and dispersed across a large number of individuals in society that it cannot be effectively collected and processed by any centralized agency (Hayek, 1948). It is the market interaction and economic reasoning of individuals guided by their own beliefs and preferences that render market prices “signals” of the underlying beliefs and preferences. In other words, the market mechanism would process dispersed information and convey it in the form of market prices. In this view, markets are conceptualized as *information processors* (Hayek, 1945). From the work of Hayek onward, information has acquired a central status among the basic conditions of market structure.

Various benchmarks can be used to assess markets. Traditionally, the four main market benchmarks are static and dynamic efficiency, equity (a particularly relevant benchmark in fields like law and economics), and macroeconomic stability (Scherer, 1980). Efficiency can be further specified depending on the scale (at the firm or at the industry level) and on the focus (technical, economic, productive, and allocative efficiency) (see Tremblay and Tremblay, 2012). Informational efficiency is the market’s property of reflecting in the prices all available information: to put it normatively, a market is informationally efficient to the extent that the market price is able to reflect all available information (Fama, 1965). In this framework, “asymmetry of information” between buyers and sellers is considered one of the main causes of market failure, i.e., when the price system does not reliably work as signal mechanism (Akerlof, 1970). Furthermore, searching for information in markets is costly (Stigler, 1961), so that the search process may considerably affect the convenience of using the market mechanism. The cost of information is one of the determinants of so-called “transaction costs,” i.e., those costs associated with the use of the market mechanism (Williamson, 1981a). The fact that transaction costs affect the convenience of the market mechanism leads buyers (individuals or firms) to look for other, possibly more convenient, coordination mechanisms. One main alternative to markets is the “make” option, i.e., buyers/firms decide to make a product or service on their own instead of buying it on the market (Coase, 1937).

Hierarchies may be preferable to the market mechanism when transaction costs are high. The existence of hierarchies – such as large-scale business enterprises – can be explained by structural features of complex and advanced economies in which transaction costs play a fundamental role (Williamson, 1981b). Chandler’s (1977) provocative notion of the ‘visible hand’ emphasizes the role of managerial activity for coordinating the allocation of economic resources as an alternative to market mechanism. Such a view deliberately contrasts with the classical view of market as an efficient and self-organizing device. But hierarchies are not the sole alternative to the market form of coordination. For instance, ‘clans’ are alternative forms to hierarchies and markets as they characterize situations in which obligations among transacting agents are not coordinated by an authority (as it happens in hierarchies) and cannot be extinguished ‘just in time’ (as it is possible in some forms of market) (see Ouchi, 1980; Adler, 2001).

Buyers and sellers, whether single individuals or firms, are said to act according to their beliefs, preferences and, most importantly, convenience. This is a basic assumption of neo-classical economics. In the last decades, the new field of ‘behavioral economics’ has tried to introduce more realistic assumptions on how agents behave in economic contexts (such as markets), by investigating the behavioral and cognitive determinants of economic actions. Nowadays, behavioral economics has become a standard framework in economics, by abandoning the aprioristic form of theorizing typical of neoclassical economics, and embracing experimental methods borrowed from the behavioral and cognitive sciences (Mullainathan and Thaler, 2000; Camerer and Loewenstein, 2004). The new partnership between economics and behavioral and cognitive psychology has rendered a more realistic image of market participants as ridden by behavioral and cognitive biases, which prevent them from reliably and consistently processing (even their own) information. For instance, the “endowment effect,” which is the difference between the price a seller assigns to her own product and the price that seller would be willing to pay, had she to buy that product on the market (Kahneman et al., 1991), arguably affects the reliability of the market mechanism (Kahneman et al., 1990). By taking this behavioral route, economic models of markets now typically include agents that are both rational (i.e., utility maximizers; see Blume and Easley, 2008) and irrational (i.e., non-utility maximizers) (Akerlof and Yellen, 1985; Russell and Thaler, 1985), or agents who are “psychologically enhanced,” i.e., provided with behavioral features (e.g., Bénabou and Tirole, 2016). Some ‘pragmatic’ interpreters of behavioral economics (e.g., Chetty, 2015) maintain that the true aim of behavioral economics should be that of discerning the contexts in which the assumptions of neoclassical economics work from those in which they should be replaced. Behavioral finance is a field that makes large use of cognitive and behavioral insights to study financial decisions beyond the neoclassical view (Barberis and Thaler, 2003). As far as markets are concerned, behavioral finance suggests that behavioral and cognitive biases would ultimately explain why prices in financial markets follow “irrational” patterns (Shiller, 2015). This does not mean, however, that behavioral economics as such supports an anti-market position (Sugden, 2018).

By taking a different route with respect to behavioral economics, also in the kind of experimental methods employed (Hertwig and Ortmann, 2001), the field of so-called ‘experimental economics’ studies how monetary incentives and rule-based coordination mechanisms are able to cancel out individuals’ cognitive biases either in single transactions or in the aggregate (Smith, 2007). The development of new game-theoretic tools has allowed economists to study how various market arrangements differ in terms of information efficiency, not just with the aim of assessing extant markets’ efficiency but also with the aim of designing from scratch new markets with customized informational properties. The possibility of designing entirely new markets stems from the development of information technologies (ITs) allowing the construction of virtual marketplaces and from the development of new branches of economics able to provide a more complex and sophisticated picture of market phenomena (Shapiro and Varian, 1998). The development of new fields in economics such as “mechanism design” (e.g., Hurwicz and Reiter, 2006), and more specifically “market design” (Vulkan et al., 2013), testifies to the fundamental need to address new forms of market, a need invariably satisfied by focusing and intervening on markets’ informational properties (Mirowski and Nik-Khah, 2017). Typically, market designers aim to off-load much of the market participants’ cognitive burden onto the rules of the market: market rules as strict, unambiguous, and as easy-to-follow as possible would lead to more effectively attain the desired level of efficiency. Analytical results that demonstrate that market efficiency can be attainable also by “zero-intelligence” traders (Gode and Shyam, 1993) support this market design’s constructivist view.

The rise of market design approaches also stems from economists’ awareness that markets are better conceptualized as “institutions” that order interpersonal relations.^1^ This view is best represented by the work of Douglass North (e.g., North, 1990). Institutions, according to North, would stem from individuals’ “shared mental models” (Denzau and North, 1994), and institutional change would take place only when these mental models change (North, 2005).^2^ The lack of perfect knowledge or information, i.e., uncertainty, would be at the root of this process of mental model sharing:

Under conditions of uncertainty, individuals’ interpretation of their environment will reflect their learning. Individuals with common cultural backgrounds and experiences will share reasonably convergent mental models, ideologies, and institutions; and individuals with different learning experiences (both cultural and environmental) will have different theories (models, ideologies) to interpret their environment (Denzau and North, 1994, pp. 3–4).

North’s emphasis on mental models testifies to the fact that this strand of research on “markets as institutions” is part of the information-processing paradigm within economics, as these mental models are mainly constituted by beliefs, preferences, expectations that populate people’s mental representations of the institutions they act in.

We can agree with Denzau and North that something is “shared” in markets conceived as institutions. We pursue this suggestion in the following sections: what is shared are the cultural practices, the external cognitive artifacts and technologies that contribute to making the market an institution, rather than internal mental variables. The notion of zero-intelligence traders is an abstraction; but there is something real that can be specified about market intelligence. One attempt to build on this kind of externalism can be found in the notion of the extended mind.

## Market as Extended Mind

Social and economic environments are more than passive products of human agency; they actively contribute to the reproduction of the stable organizations and practices that enable and constrain human behaviors (see Giddens, 1984). Accordingly, markets are more than inert structures for information processing devoted to solving allocation and coordination problems involving collectivity. Borrowing significantly from Denzau and North, Clark (1997a) conceives of markets as structures that provide epistemic scaffolding, involving strong constraints and incentives that predictably direct agents’ behavior. Such structures are able to produce a “cognitive economy” as they steer individuals’ decisions and actions: they reduce in a significant manner the cognitive effort for information processing by externalizing a number of processes. The idea that economic decision-making takes place in such highly scaffolded environments would explain why neoclassical economics works, “(insofar as it works at all)” (Clark, 1997b, p. 271).

Clark understands scaffolded cognition as an instance of the extended mind (Clark and Chalmers, 1998). The idea of the extended mind is based on the general hypothesis that cognitive processes are not limited to what happens in the head but may occur by allowing the external world to do some of the work. Factors external to brain and body may be functionally integrated in the overall cognitive system. On this account, cognition consists of a specific kind of action that manipulates an external tool or instrument, for example, using pencil and paper to do math. Such extension occurs in cases in which the manipulation of the external world can be considered functionally equivalent to internal processes (Clark and Chalmers, 1998). What allows something to be part of a cognitive system or “a proper part of a genuinely cognitive process” (Clark, 2010, p. 85) is tied to its function.^3^ According to the extended mind hypothesis, this is expressed as the “parity principle” and is stated as follows.

If, as we confront some task, a part of the world functions as a process which, were it done in the head, we would have no hesitation in recognizing as part of the cognitive process, then that part of the world is (so we claim) part of the cognitive process (Clark and Chalmers, 1998, p. 8).

Doing math in one’s head counts as a cognitive process; likewise, doing it with paper and pencil should count as a cognitive process where paper and pencil function as a mechanism or vehicle of cognition, functionally similar to internal (e.g., neural) mechanisms. Just as in our heads we may manipulate a mental model to solve the problem, we manipulate paper and pencil to accomplish the same task (Clark, 1997b, p. 297).

If in some cases there is a functional similarity between inner and outer processes, there are also many cases that involve significant differences. This motivates an emphasis on complementarity or functional integration, which includes the idea that “different components of the overall (enduring or temporary) system can play quite different roles and have different properties while coupling in collective and complementary contributions to flexible thinking and acting” (Sutton, 2010, p. 194; see Menary, 2013). Functional integration is indexed by differences in individual cognizers, and differences in particular environments. Individual agents may have different proclivities to use external props and instruments versus internal processes like memorization, and this balance might be modulated by changes or structures in the environment or what one particular environment in contrast to another affords.

The argument for the extended mind thus turns primarily on the way disparate inner and outer components may co-operate so as to yield integrated larger systems capable of supporting various (often quite advanced) forms of adaptive success (Clark, 1997b, p. 99).

The ideas of parity and complementarity signal, respectively, different ways in which internal processes (beliefs, desires, mental models and other representational states) play a role together with the external vehicles or mechanisms that scaffold cognition. The extended mind involves a hybrid of internal and external processes where, in some cases, the cognitive processes are carried primarily by external factors, and in others, by internal factors.

Building on the work of Denzau and North (1994) and Satz and Ferejohn (1994), Clark recognizes that the larger system supporting cognition can include institutions. “Institutions, firms, and organizations seem to me to share many of the key properties of pen, paper, and arithmetical practice in this example” (Clark, 1997b, p. 279). At the same time, such institutions impose structural limitations on individual choice. “[W]hat is doing the work, in such cases, is not (so much) the individual’s cogitations as the larger social and institutional structures in which she is embedded” (Clark, 1997b, p. 272).

In terms of economics, Clark’s argument continues, market mechanisms understood as institutional rules and practices, promote actions that maximize returns relative to a fixed set of goals. Thus, “firms and organizations provide an external resource in which individuals behave in ways dictated by norms, policies, and practices; norms, policies, and practices that may even become internalized as mental models” (Clark, 1997b, p. 279). In many cases, rather than basing economic choices solely on a set of beliefs, desires, or other psychological states within the individual, larger scale market structures that rule firm-level strategies impose strong constraints on individual choice.

In the embrace of such powerful scaffolding, the particular theories and worldviews of individuals may at times make little impact on overall firm-level behavior. Where the external scaffolding of policies, infrastructure, and customs is strong and (importantly) is a result of competitive selection, the individual members are, in effect, interchangeable cogs in a larger machine. The larger machine extends way outside the individual and incorporates large-scale social, physical and even geopolitical structures (Clark, 1997b, p. 272).

Individuals may play interchangeable functional roles [at the extreme as zero-intelligence traders (Gode and Shyam, 1993)] in the larger institutional processes. Such processes may be underdetermined and open to varying dynamics of positive feedback (the result of, for example, small early perturbations in the overall economic system) (Arthur, 1990), but these effects may still involve external factors rather than individual psychological determinants. Accordingly, “the explanatory burden is borne by overall system dynamics in which the microdynamics of individual psychology is relatively unimportant” (Clark, 1997b, p. 276). This, according to Clark, but also according to the thought of well-known neoclassical economists (e.g., Becker, 1962), would ultimately explain why neoclassical economic theory works:

In cases where the overall structuring environment acts so as to select in favor of actions which are restricted so as to conform to a specific model of preferences, neoclassical theory works. And it works because individual psychology no longer matters: the “preferences” are imposed by the wider situation and need not be echoed in individual psychology (Clark, 1997a, p. 183)

This does not mean that there is no role for individual psychology in the economic system. Clark brings us back to the notion of the extended mind where individual cognizers are coupled to various external factors and institutional practices. Specifically, there is interplay between individual internal mental processes and a set of larger mechanisms that accounts for innovation, the possibility of learning and expanding intellectual horizons. Clark suggests that these may depend on individual idiosyncrasies and positive feedback effects (Clark, 1997a, pp. 186–192). We think there is more to say on this issue. At any point beyond zero-intelligence, the larger mechanisms can support and motivate different practices leading to unpredictable innovation outcomes. One type of behavior allowed but not strictly dictated by the market mechanism is, for instance, strategic behavior.

It would be useful for purposes of explicating Clark’s views further, to see how both sides of the markets, suppliers and consumers, can use the market mechanisms in seemingly ‘unorthodox’ ways. In general, consistent with Clark’s framework, price can be re-conceptualized as an *offloading* device, i.e., a *cognitive artifact* (see Risko and Gilbert, 2016), able to reduce cognitive demands. Consistent with Clark (2005)’s emphasis on the external vehicles of cognition, prices can be considered external artifacts able to compensate for the lack of information about allocation possibilities, which can be considered part of economic agents’ bounded rationality (e.g., Simon, 1982; see also Arnau et al., 2014).^4^ Clark, however, suggests that the market mechanism differentially affects market participants. On the supply side, he emphasizes that “[s]trong constraints imposed by the larger scale market structure result in *firm-level strategies and policies* that maximize profits” (Clark, 1997a, p. 272, emphasis added). In other words, suppliers would be highly scaffolded, to the point of being induced to maximize profits (or, more generally, utility), as dictated by neoclassical economics.

Even if evidence on bounded rationality largely shows that firms do not systematically maximize profits (Simon, 1979), we suggest there is an even more important point to consider, namely, that imposing constraints is not the only direction in which a market scaffolds suppliers. For example, suppliers can use the price mechanism strategically. Such strategic use of the market can be visible, for instance, in the implementation of price strategy – to defend market positions or conquer new segments, for example – as prices can be considered signaling and explorative tools (Spiegler, 2011; Tremblay et al., 2018). This does not imply that strategy is the only way in which the market enables suppliers. A variety of suppliers’ behaviors are enabled by the market mechanism that are not necessarily compatible with the immediate purpose of maximizing profits. This is to say that scaffolding works in two directions and not only in the constraining way (see also Cardinale, 2018). There are degrees of freedom in the scaffolding mechanism, and the very same market mechanism can enable novelty. In this view the same notion of market efficiency, discussed above, is challenged by the consideration that the economic environment continuously invites new uses of existing economic resources, allowing the emergence of innovations (Felin et al., 2016).

On the demand side, Clark claims that “the theory of consumer behavior is weak […] and the external scaffolding is commensurably weaker” (Clark, 1997a, p. 183), thus implying that consumers would be less bounded by the market mechanisms than suppliers. In our understanding, however, this postulated asymmetry between supply and demand can be misleading. We know that consumers, by definition, go to the market in order to solve their problems to procure their means of subsistence compatibly with their budget constraints. Even this simple neoclassical characterization of the consumer problem would suffice to make clear that consumers are, in principle, as equally scaffolded as suppliers by the market mechanism. Even if we are less surprised that consumers often behave more irrationally than suppliers do (but, not to forget, also suppliers can be irrational, see Kahneman et al., 1991), again this may not be the full story. The other part of the story is the central and active role played by consumers in the market mechanism, which goes beyond the simplistic view that markets pragmatically provide goods and epistemically provide information. Leibenstein (1950) emphasized that there are various motives behind consumers’ actions in the market (such as functional, speculative, social, and irrational motives). We may add that there exists a further motive, in so far as by acting in the market the consumer behaves so as to solve a real epistemic problem. Not only does the consumer’s behavior (“to buy” or “not to buy” at a certain price) contribute to determining the market price; in addition the market process serves to extend the consumer’s cognitive process in order to solve the consumer’s problem, which involves reliably attaining a correct evaluation of the good.^5^

We are led to the following ideas: markets (a) emerge from the interaction of both sides (supply and demand), in their continual and mutual interplay, and (b) involve more basic social interactions that shape both sides. In both cases – supply and demand – actions involved in price negotiations (in terms of bid and ask prices) can be considered from an epistemic point of view, not just pragmatically. Kirsh and Maglio (1994 p. 513) distinguish between pragmatic and epistemic actions: pragmatic actions are “performed to bring one physically closer to a goal” whereas epistemic actions are “performed to uncover information that is hidden or hard to compute mentally.” In these terms, price setting can be considered not just as a pragmatic action that puts market parties a step closer to the exchange moment, but as an action in which prices as artifacts are manipulated for epistemic reasons, that is to say, to uncover, or even create new knowledge. For example, on the supply side a firm could change prices to evaluate how their competitors will react to different price levels, and in general to try to discover their strategic intent. On the demand side, a consumer could negotiate the price not only to save money but to create a stable and credible contact with the seller. To some extent, negotiating could be a way to disclose intentions, and establish trust and reciprocity between parties.

## Market as Cognitive Institution

In this and the next section, we build upon the notion of the market as a “scaffolding institution” understood in terms of the extended mind. We pursue an enactivist interpretation that emphasizes social interaction. Thus, we propose to understand a market as a socially extended cognitive institution and to develop a picture of economic interactions among agents framed in terms of relational autonomy, in an economic order enacted by those very same economic agents. That markets are cognitive institutions enabling and constraining economic reasoning also leads to the possibility of specific types of economic reasoning processes.

A market is not just a mechanism, a structure, or a narrowly-conceived institution; it’s a social institution, and it emerges as such because it involves intersubjective interactions embedded in social and cultural practices. In an extended-mind model of scaffolded choice, at least in some cases, rather than “beliefs, desires, or other psychological features of individuals involved,” what counts are the external structures that constrain and enable economic agents’ behavior (Clark, 1997b, p. 272; see also Denzau and North, 1994). Taking this one step further, on the model of a *socially* extended mind (Gallagher, 2013) the constraints imposed by social interactions, as well as the possibilities enabled by such interactions, are such that *economic reasoning* is never just an individual process carried out by an autonomous individual, classically understood. Such considerations change the theoretical notion of market from a mere economic mechanism able to solve allocation and coordination problems of collectivity (in specific circumstances, better than alternative mechanisms) to an enactive cognitive institution. Considering the perspective of the agents involved, the market as *cognitive institution* is like Clark’s extended mind notion of scaffolded cognition insofar as it (i) extends the participants’ cognitive processes of economic reasoning, and (ii) both constrains and enables the actions and interactions of embodied and embedded agents in the economy. The enactive notion of cognitive institution involves something more, however.

To see this, consider Slors’ (2019) recent clarification of the difference between an extended-mind conception of institution as a causal-functional unit, and the enactive model of socially extended cognition, i.e., the idea of a mental or cognitive institution. Slors defines cognitive institutions, following Gallagher (2013, p. 6): “not only as institutions with which we accomplish certain cognitive processes, but also… without [which] such cognitive processes would no longer exist.” Socially extended cognition is constituted in a specific form of dynamical engagement with the world, one that involves reciprocal causality.^6^ Simply put, an institution is formed by cognitive (e.g., problem solving) practices that involve multiple interacting agents pursuing multiple interrelated tasks, and reciprocally, such interactions are shaped by instituted (normative) practices that extend our cognitive processes when we engage with them (that is, when we interact with, or are enactively coupled to them in the right way).

This includes, as an example, the legal system, which “enables an array of thoughts and actions that are unintelligible without the concepts and procedural social routines associated with the law” (Slors, 2019, p. 5). The practice of law is constituted by just such cognitive and communicative processes carried out in the cooperative activities of many agents relying on conventional cognitive schemas and rules of evidence provided by the legal institution itself. Reasoned judgments made in such contexts, specified as *legal* judgments precisely because they are made in such contexts, are forms of cognition that depend on the large and complex system without which they could not happen. For example, in the case of a highly trained attorney who may be engaged in a process of legal reasoning, what makes this kind of cognition what it is depends not only on the fact that she was trained within a related institutional system (i.e., in the specific practices of law school), but also on the continued workings of the legal system. Indeed, some tasks would never even arise if it were not for the legal system.

Slors contrasts the extended-mind conception of institution, which, as we saw in the previous section, is based on the idea of functional integration, with what he calls a “symbiotic” arrangement. He argues that in contrast to Clark’s concept of institution (derived from Denzau and North, 1994) – understood as an external mechanism that structures and orders the individual’s environment so as to scaffold cognition – the symbiotic cognition model is a better way to think about socially extended cognitive institutions. A cognitive institution is different, in principle, from the pencil and paper that I might use to solve a math problem. Specifically, Slors defines the notion of symbiotic cognition in terms of “task dependency.”

“Task dependency” is the extent to which the intelligibility of a task depends on a larger whole of coordinated tasks. Task dependency is a notion that is connected with coordination and planning. It is a normative notion in the sense that high task dependency means that tasks play specific roles in the overall organization of a cognitive system or a cultural cognitive ecosystem; roles that can be played properly or improperly (Slors, 2019, p. 18).

For example, the legal system is characterized by high task dependency since judge, prosecutor, defense attorney, clerk, and other officials are inter-defined in a holistic way, such that what an attorney does is understandable only by referring to what judges and prosecutors do. As Slors suggests, this means that there is a division of labor in a symbiotic system.

Division of labor involves a specific type of offloading, one which is typical for symbiotic cognition but not for extended [mind]. Every participant in a symbiotic system profits from whatever the system as a whole offers (education, justice, social coordination) while contributing only a small part. The tasks, jobs and roles of others in the system co-define and enable one’s own task, but one does *not* have to perform them or even think about them, while nevertheless benefiting from the overall outcome of the system (Slors, 2019, p. 30).

In regard to the concept of market, we suggest that on a symbiotic model one would have to think about market dynamics in more complex terms than simply supply side and demand side.^7^ On the symbiotic view, the market is a “marketplace” (as Callon, 1998 specifically defines it) – that is, a real set of human interrelationships embedded in a workspace of different tasks – government regulator or planner, corporation, manufacturing unit, information (or other service) provider, marketer, wholesaler, retail agent, purchaser, consumer (household), and any number of economic roles in between these categories. Each task category may be defined not simply by economic principles, but by non-economic norms and practices, and by less formal and imperfect social interactions that may involve a variety of biases. Different task-players are dynamically related in a gestalt arrangement such that an intervention (above a certain threshold) on one node or element in the system will lead to modulations in other nodes or elements, or in the whole (Gallagher, 2018b).

The concept of symbiotic arrangements clearly characterizes some forms of cognitive institutions, but we note that, as Slors acknowledges, the contrast between functional integration and task dependency is a matter of degree. He suggests that the legal system is characterized by high task dependency and low functional integration, and he then (perhaps too quickly) generalizes this to apply to all cognitive institutions in contrast to extended mind models (high functional integration; low task dependency), and models of distributed cognition (high functional integration; high task dependency). We think the issue is more complex and that the distinction between “low” and “high” functional integration and task dependency is probably too coarse-grained; rather, cognitive institutions vary in degree between task dependency and functional integration depending on where one is looking in the system, or from what perspective one examines the system.^8^ For example, in the legal system, from a systems perspective one sees high task dependency, whereas from the perspective of the individual agent who engages with the system, one finds a significant degree of functional integration. An attorney, for example, has to make the system work by doing certain things that require material engagement with papers, law books, courtrooms, and many other people. What she does may be defined in terms of specific tasks, but those tasks are accomplished only by engaging with instruments and people, and often in flexible and creative ways. Contracts and written (official) documents are instrumentally functional and, at the same time, they are “pieces” of the legal structure that in some cases predefine or scaffold the roles of individuals. That is, at the same time, they are, from the individual’s perspective, functionally instrumental for extending legal reasoning and, from the systems perspective, constitutive parts of the legal structure.^9^

We propose that more generally a cognitive institution always involves varying degrees of task dependency and functional integration. A market system is a good example of a cognitive institution in this regard. A market is symbiotic, not in Slors’ sense, where the level of functional integration is low, but in the sense that there is always a co-dependency between the actions and social interactions of individual agents and the market institution. Buying or not buying a good in the market is, from an interactive point of view, something that enacts the market. In this respect, the level of functional integration is high. Engaging in “epistemic actions” (biding or selling items on the market) enacts the market such that the market would not be there without these actions. At the same time, the market, as a cognitive institution is not only an institution that supports or scaffolds specific acts of economic reasoning; it is also such that without it “such cognitive processes would no longer exist” (Gallagher, 2013, p. 3). From this perspective, markets and price mechanisms are more than extended processes that scaffold economic reasoning about the scarcity of goods. They are institutions for *enacting* economic, task-dependent relations, as social interactions, which themselves become the object (or subject-matter) of economic reasoning, *which would not exist – as we know it – without markets*.

Specifically, we contend, there are reciprocal relations, symbiotic interactions involved in the cognitive institution, characterized by degrees of both functional integration and task dependency. The judge not only extends his cognitive processes by engaging with the legal system through, or facilitated by, a set of intersubjective interactions; in addition, it’s precisely by the judge’s engagement (and many other such engagements) that the legal system is enacted. Just so, the individual economic agent extends his reasoning by engaging with the market through, or facilitated by,^10^ a set of intersubjective interactions, thereby epistemically benefiting from the market process; and reciprocally it is precisely by that engagement (and many other such engagements) that the market is enacted.

The efficiencies and inefficiencies, as well as degrees of trust and mistrust (to make room for what Clark (1997a, p. 276) calls “individual psychological profiles,” without putting them entirely back in the head) present in the market are anchored in the specific types of social interactions that a market makes possible. It’s true, as Denzau and North (1994) point out, that gains from trade and productive coordination in a market economy are based on the existence of some degree of trust: “The morality of a business person is a crucial intangible asset of a market economy, and its non-existence substantially raises transaction costs” (Denzau and North, 1994, p. 20). Trust, however, to whatever degree, is not the product of shared mental models, as they suggest; it’s a product of and varies with different types of intersubjective interactions; it gets cashed out in the meaning that emerges from and transcends any individual’s actions or thoughts as it gets instituted (De Jaegher et al., 2010).

The trust that is characteristic, for instance, of impersonal (typically electronic) forms of financial markets is not equivalent to forms of intersubjective trust that may characterize hierarchical or clan societies. In impersonal (e.g., anonymous) markets, economic reasoning, and appropriate degrees of trust, are enabled by the fact that individual decisions may sometimes be “cold” and calculative since an interaction with other economic agents may be “living” only in the immediate transaction, through which a mutual benefit is reached and after which all obligations are extinguished. Price mechanisms allow an efficient allocation so that before and after the transaction all information is exploited and balance is immediate. Of course, we are not arguing that this is the case of any market (many real-world markets require the building of long-term relationships, see Ben-Ner and Van Hoomissen, 1991), but that some markets are specifically appreciated for their frugality and impersonality. In other forms of institution (such as hierarchies or clans) the obligations between parties typically persist in the long-term. Accordingly, the single transaction in itself is not necessarily fair. In the case of a clan, the relational dynamics involved are something like “I do something for you today, and you will do something for me in the future;” in the case of hierarchies: “you work hard now but in the future you will be promoted to manager.” Balance is postponed. Economic reasoning varies across these different institutions precisely because interactions and trust relations vary.

## Critical Implications: The Reification of Relational Potential

Adam Smith’s works *The Theory of Moral Sentiments* (Smith, 1759) and *The Wealth of Nations* (Smith, 1776) represent two significant contributions to understanding the origins of markets and their institutionalization. While in the first work Smith emphasizes the importance of “sympathetic” interaction for the development of morality, in the second he emphasizes the importance of self-interest and the calculative attitude as requisite features for a proper functioning of market economies. The intrinsic unity of the two contributions has hardly been acknowledged by neoclassical economics (see Bruni and Zamagni, 2007, Chapter 5), so much so that it has usually been easier to postulate the existence of two different Smiths (Smith, 1998). What results as problematic from the partial reading of Smith’s opera is a notion of autonomy, understood in terms of self-sufficiency, self-legislation, or self-determination, which has colonized economics in general (Nelson, 2006), and the economics of market in particular (Zak, 2008; Sandel, 2013), through the notion of *homo oeconomicus.* This situation does not particularly change if we add more modern readings of Smith’s work, which for instance identify Smith as a father of behavioral economics (Ashraf et al., 2005).

An alternative concept of *relational autonomy* is based on the idea that autonomy is actualized in social interactions that involve varying and imperfect degrees of mutual recognition (Mackenzie and Stoljar, 2000; Honneth, 2008; Gallagher, 2017). On an enactivist view of social interaction there is always a balanced and partial trade-off between the autonomy of the individual embodied agent and the autonomy of the process of social interaction itself (De Jaegher et al., 2010). Interaction requires the preservation of some degree of individual autonomy, but that makes one’s autonomy relative to other agents and to the nature of specific interactions. Autonomous actions are thus embodied and situated in a world that is physical and social. This interpretation correlates with a more enactive view of economic reasoning that understands the market participant in terms of social interactive processes, and an autonomy that is by degree and that exists for the individual agent only because she is socially situated. Economic reasoning, understood in terms of relational autonomy, is always (to varying degrees) embodied,^11^ embedded in material engagements, and scaffolded by intersubjective interactions. By contrast, the relational dimension is so much absent in neoclassical economics that, in economic modeling, it is often sufficient to assume the existence of a “representative agent” to stand in for the collectivity (Kirman, 1992).

But markets can be a double-edged sword. A market operating as a cognitive institution – enabling and constraining economic reasoning – can easily reflect an ideology. Market ideology is probably a byproduct of markets as cognitive institutions. There are reasons to think that markets – and in particular electronic financial markets, which can be viewed as market forms engineered to facilitate impersonal coordination – in some cases undermine recognition and relational autonomy, by imposing a form of “avatar recognition,” a reification in which one’s self and others are reduced to merely rational/calculative agents. Reification “means a forgetting of the primal recognition that two humans accord each other in a fundamental process of intersubjective interactions” (Jay, 2008, p. 8; see Honneth, 2008). In other words, reification is the opposite of autonomy.

Reification and the denial of autonomy, are real phenomena at the political level of nations and subnational groups, but they can be just as real in our everyday lives, in our relations with others, as well as in the externally imposed bureaucratic, administrative, and institutional pathologies that Honneth points to as involving “cold” and “calculating compliance” (Honneth, 2008, p. 17). Reified and pre-packaged ways of interacting lack dynamic spontaneity, impose a mechanistic order, and can undermine the autonomous processes implicit in genuine forms of interaction. It is important to note, in this regard, how reification can be even counterproductive with respect to economic principles themselves. Bowles (2016) makes a compelling case that market design and incentive-based policies are not the “substitute of good citizens.” As Adam Smith acknowledged, non-strictly-economic values are needed to make a market really function (see also Zak, 2008). When the neoclassical notion of economic rationality is detached from more basic social interactions, it becomes a self-fulfilling prophecy (see Denzau and North, 1994; Ferraro et al., 2005), in so far as markets can be engineered in view of programmatically fostering and selecting that sort of rationality. In other words, markets can be said to be “performed” by economic theory, which would shape markets in its own image by imprinting in them its own notion of rationality (Callon, 1998; MacKenzie, 2006).^12^ Markets are ways in which we can reasonably understand and predict others’ behaviors within our human interactions; at the same time, the fact that we can do this means that often the relational potential is sometimes reified to calculative purposefulness.

Honneth (2008, p. 24) describes a change of perspective from empathic/sympathetic engagement to detached observation. The latter tends toward a reification of others and can be found in attitudes that commodify relationships and interaction (e.g., Summerville and Chartier, 2013). Reification and commodification can even become, as it has been observed in particular by Marxist theorists with special reference to the large mechanisms of capitalism, a social practice strategically relevant to social struggle (Postone, 1993). Honneth (2008, p. 28) however, suggests that the detached, observational relation may in fact be a necessary strategic stance required in developed societies to deal with some aspects of the business of everyday life. This kind of detached stance may have a “perfectly legitimate place” in some situations. How legitimate market detachment is also crucially depends on the object of the transaction [think of markets for organs from living donors (e.g., Rippon, 2014), which could be viewed as a patent example of reification (see Satz, 2010)]. Still, we can ask to what extent a market structure, intended as the set of market features that enable/constrain market participants, contributes to such detached attitudes and reductions in relational autonomy. That is, in studying the way that markets work, we can recognize the variability of market institutions in terms of how they affect intersubjective recognition and autonomy, thereby giving us a way to ask critical questions about how they might be adjusted or transformed with a view to reducing reification, increasing autonomy, and addressing institutionally generated distortions in intersubjective interactions.

## Conclusion

An important part of what it means for agents to be situated in the everyday world of human affairs includes their engagement with economic practices. Traditional economic theory views the market as a set of mechanisms for exchange and allocation or coordination. Such conceptions focus more on the *structure* of markets and how information is processed by market structure in ways that may facilitate the deployment of a set of mental models and behaviors by the individual participant. This leads to the specific idea of market institution as external mechanism that orders or structures rational decisions and human relations. These ideas are taken up by Clark and framed in terms of the extended mind hypothesis. In this case, the market is understood as scaffolding economic reasoning *via* strong constraints that direct agents’ behaviors in predictable ways. Markets produce a “cognitive economy” by reducing individual cognitive effort thereby steering individuals’ decisions and actions.

We’ve argued that a market understood as an institution is not just a mechanism, or an external structure narrowly conceived; rather, it’s a social institution that emerges as such from intersubjective interactions in social and cultural practices. On this view, it not only extends the participant’s economic reasoning processes, constraining and enabling the actions and interactions of embodied and situated agents in the economy, but as a cognitive institution it is enacted in just these processes and is characterized by varying degrees of both task dependency and functional integration.

An enactive perspective on the market as a socially extended cognitive institution offers a picture of the economic interactions of individuals framed in terms of relational autonomy, in an economic order enacted by those very same economic agents. That markets are cognitive institutions enabling and constraining economic reasoning also leads to the possibility of specific types of economic reasoning processes, characterized in some cases by calculative purposefulness. Speculatively, this latter form of economic reasoning can be considered a “materialization” of Weber’s ideal-type of “*Zweckrationalität*” or instrumental rationality. In the extreme it can lead to distorted social interactions. This is clearly recognized by critical theorists when they inquire about how institutions shape both our cognitive processes and our interpersonal interactions (Honneth, 2012; Gallagher, in press). An understanding of real-world markets as socially extended cognitive institutions helps us to see that designing and performing markets in ways that might counter distorting modes of rationality are not simply about changing external structures, but can have an effect on the individual (relational) autonomy involved in everyday situated practices.

## Author Contributions

All authors listed have made a substantial, direct and intellectual contribution to the work, and approved it for publication. Authorship is listed alphabetically.

## Conflict of Interest Statement

The authors declare that the research was conducted in the absence of any commercial or financial relationships that could be construed as a potential conflict of interest.

## Footnotes

1 On the basis of North (1990), Denzau and North (1994, p. 4) define institutions as “the rules of the game of a society [consisting] of formal and informal constraints constructed to order interpersonal relationships.”

2 “The mental models are the internal representations that individual cognitive systems create to interpret the environment; the institutions are the external (to the mind) mechanisms individuals create to structure and order the environment” (Denzau and North, 1994, p. 4).

3 The details of the relationship between functionalism and the extended mind have been a focus of a number of important papers – see Sprevak (2009), Miyazono (2017), and Wadham (2016).

4 As Clark suggests: “Simon saw, very clearly, that portions of the external world often functioned as a non-biological kind of memory. He thus saw the deep parity (parity, not identity) that can obtain between external and internal resources” (Clark, 2001, p. 139).

5 For a different sort of epistemic problem solved by markets consider the case of so-called “prediction market,” i.e., market used to reliably predict future events (e.g., Wolfers and Zitzewitz, 2004).

6 The notion of constitution at stake in this concept of socially extended cognition is not simply, as Slors suggests, the notion of a synchronic compositional constitution, as one finds in the new mechanist literature (e.g., Craver, 2007), but also involves a diachronic dynamical constitution understood to involve reciprocal causal relations. Acknowledging the concept of dynamical constitution is a way to avoid one of the major objections to the extended mind idea – the idea that extended and enactive models commit a causal/coupling-constitution fallacy (Adams and Aizawa, 2008; Aizawa, 2010, Aizawa, 2014, and Gallagher, 2018b).

7 This is what economic sociologists typically do (e.g., Burt, 1992; Fligstein, 2001).

8 We take the distinction between task-dependency and functional integration to reflect different kinds of coupling. Task-dependency involves structural coupling (an agent engages with the system by performing a certain type of task or occupying a certain place in the system); functional integration is causal (or dynamical, reciprocally causal) coupling. One can characterize different cognitive systems (or institutions) as involving different combinations of these kinds of coupling.

9 Slors further points out that in a symbiotic system the interaction that constitutes a cognitive institution “is facilitated by a physical infrastructure and specific physical artifacts.” Although this is clearly an aspect that fits with conceptions of extended minds and markets, we note that material engagement theory (Malafouris, 2013) leads to a stronger claim based on enactivist principles: markets are constituted by material engagements which in turn, and over time, shape the rationality and agency of market participants and create meanings that go beyond economic significance. This involves not only engagement with commodities – goods or services that depend on production or exchange facilities, infrastructure, transport and communication equipment, as well as advanced technology processes – but also a range of embodied or virtual intersubjective interactions that characterize any institution. There is much more to say on this point, but to pursue it would lead beyond the scope of this paper.

10 That the engagement is “facilitated by” a set of intersubjective interactions is a way of expressing the idea that even in cases of anonymous activity where there is no occurrent intersubjective interactions (as, e.g., in regulated financial markets or in computer-run algorithmic trading) the anonymous processes are ultimately grounded in previous intersubjective interactions.

11 For economic reasoning as a form of embodied rationality see Mastrogiorgio and Petracca (2016); also Gallagher (2018a); for traditional forms of rationality in economics see, e.g., Blume and Easley (2008).

12 Similar criticism has been made about the large (and often reductionist) claims about human nature sometimes made by neuroscience, and especially popular media coverage of neuroscientific discoveries, namely that we all start to think of ourselves in those reductionist terms. See, e.g., Choudhury and Slaby (2012) and Slaby (2010).
